# Epidemiology of infections by HIV, Syphilis, Gonorrhea and Lymphogranuloma Venereum in Barcelona City: a population-based incidence study

**DOI:** 10.1186/s12889-015-2344-7

**Published:** 2015-10-05

**Authors:** Marc Martí-Pastor, Patricia García de Olalla, Maria-Jesús Barberá, Christian Manzardo, Inma Ocaña, Hernando Knobel, Mercè Gurguí, Victoria Humet, Martí Vall, Esteban Ribera, Judit Villar, Gemma Martín, Maria A. Sambeat, Andres Marco, Alvaro Vives, Mercè Alsina, Josep M. Miró, Joan A. Caylà, and the HIV Surveillance Group

**Affiliations:** Epidemiology Service, Agencia de Salut Pública de Barcelona, Pl. Lesseps, 1, 08023 Barcelona, Spain; Teaching Unit of Preventive Medicine and Public Health, PSMAR-UPF-ASPB, Barcelona, Spain; Consorcio de Investigación Biomédica en Red de Epidemiología y Salud Pública (CIBERESP), Barcelona, Spain; Infectious Diseases, Hospital Vall de Hebron, Universitat Autònoma de Barcelona, Barcelona, Spain; Hospital Clinic-IDIBAPS, University of Barcelona, Barcelona, Spain; Infectious Diseases, Hospital del Mar, Barcelona, Spain; Infectious Diseases Unit, Hospital de la Santa Creu i Sant Pau, Universitat Autònoma de Barcelona, Barcelona, Spain; Departament de Justicia, Direcció General de Serveis Penitenciaris i de Rehabilitació, Barcelona, Spain; Fundació Puigvert, Barcelona, Spain

**Keywords:** Sexually transmitted diseases, HIV, Syphilis, Gonorrhea, Lymphogranuloma venereum, Sexual behavior, Epidemiology, Incidence

## Abstract

**Background:**

The aim of this study was to determine the evolution of HIV infection, gonorrhea, syphilis and lymphogranuloma venereum (LGV), and their epidemiological characteristics in Barcelona city.

**Methods:**

Population-based incidence study of all newly occurring diagnoses of HIV infection, syphilis, gonorrhea and LGV detected in Barcelona between January 2007 and December 2011. A descriptive analysis was performed. The annual incidence rates per 100,000 inhabitants were calculated by sex, sexual conduct and educational level. To estimate global sex-specific rates we used the Barcelona city census; for the calculation of rates by sexual conduct and educational level we used estimates of the Barcelona Health Interview Survey. Trends were analysed using the chi-squared test for linear trend.

**Results:**

*HIV*. 66.8 % of the HIV cases were men who had sex with men (MSM). The incidence rates in MSM over the study period were from 692.67/100,000 to 909.88/100,000 inh. *Syphilis*. 74.2 % of the syphilis cases were MSM. The incidence rates in MSM were from 224.9/100,000 to 891.97/100,000 inh. and the MSM with a university education ranged from 196.3/100,000 to 1020.8/100,000. *Gonorrhea*. 45.5 % of the gonorrhea cases were MSM. The incidence rates in MSM were from 164.24/100,000 to 404.79/100,000 inh. and the MSM with university education ranged from 176.7/100,000 to 530.1/100,000 inh.. *Lymphogranuloma venereum (LGV)*. 95.3 % of the LGV cases are MSM. The incidence rates in MSM were from 24.99/100,000 to 282.99/100,000 inh. and the MSM with university education ranged from 9.3/100,000 to 265/100,000 inh.

**Conclusion:**

An increase in cases of STI was observed. These STI mainly affected MSM with a university education. Continuing to monitor changes in the epidemiology of STI, and identifying the most affected groups should permit redesigning preventive programs, with the goal of finding the most efficient way to reach these population groups.

## Background

Over the last decade an increase has been observed in sexually transmitted infections (STI) in various Western countries [1–3]. In these countries, the majority of cases occur in metropolitan areas, men who have sex with men (MSM) being the group most affected [4–6].

The rise in STI has been influenced by the increase in risky sexual conduct (increase in number of partners, and subgroups engaging in more risky sexual practices) [7, 8], probably resulting from a relaxation in practising safe sex because of a false sensation of safety due to the existence of highly active antiretroviral therapy (HAART). Furthermore, HIV infection is associated with other STI, since they increase the risk of acquiring and transmitting HIV [2, 8, 9].

The aim of this study was to determine the evolution of HIV infection, gonorrhea, syphilis and lymphogranuloma venereum (LGV), and their epidemiological characteristics in a large city.

## Methods

Barcelona is a city of 1,615,985 inhabitants [10] in which the obligatory notification of syphilis, gonorrhea and LGV has been nominal since 2007, and notification of HIV infection, voluntary between 2001 and 2009, has been obligatory and nominal since 2010 [11].

This is a population-based incidence study of all newly occurring diagnoses of infection by sexually transmitted HIV, syphilis, gonorrhea and LGV detected by the epidemiological surveillance system among residents of the city of Barcelona between January 2007 and December 2011. Cases of vertical and intravenous transmissions of HIV infection were excluded, as were cases diagnosed with AIDS, and cases of late latent, terciary and congenital syphilis. Doctors who diagnosed a case completed a questionnaire which collects sociodemographic and clinical variables: sex, date of birth, country of birth ((1) Spain; (2) Western Europe, United States, Canada and Australia; (3) Eastern Europe; (4) Latin America; (5) Other) and sexual conduct ((1) MSM; (2) men who only have sex with women (MSW); and (3) women who have sex with men (WSM)). Educational level ((1) no studies; (2) primary studies; (3) secondary studies; (4) university studies), number of sexual partners in the last 12 months and use of condom in most recent sexual contact were collected in cases of syphilis, gonorrhea and LGV, but not in cases of HIV infection.

A descriptive analysis was performed, calculating annual incidence rates per 100 000 inhabitants, by sex, sexual behaviour category and educational level. In order to estimate sex-specific rates we used population figures from the Barcelona city local census [10]; for the calculation of rates by sexual conduct and educational level we used estimates of the MSM population (5.3 %) obtained through the Barcelona Health Interview Survey, along with their 95 % confidence intervals (CI) [12]. Trends were analysed using the chi-squared test for linear trend.

All analyses were peformed using the SPSS statistical package, version 18 [13]. HIV, syphilis, gonorrhea and LGV are mandatory notifiable infections for health professionals in compliance with Article 13 of the law 67/2010 (25^th^ May 2010) of the Health Department of Generalitat de Catalunya. All data was handled maintaining strict confidentiality according to the ethical principles of the Helsinki Declaration of 1964 revised by the World Medical Organization in Edinburgh, 2000 and Law 15/1999 of Data Protection in Spain [14].

## Results

### HIV

A total of 1560 cases were detected, of whom 89 % (*n* = 1388) were men, and of these 79 % (1096) declared themselves to be MSM (7.3 % of all MSM had sex with both males and females and 92.7 % had sex with men only). MSM had a mean age of 34.7 years (SD:8.7), WSM a mean age of 36.6 years (SD:9.9) and the MSW of 38.6 years (SD:12.3), differences between groups being statistically significant (*p* < 0.001) (Table 1). Regarding country of birth, 50 % (*n* = 548) of the MSM, 58.3 % (*n* = 133) of the MSW and 50 % (*n* = 86) of the WSM had been born in Spain (Table 1). However, the highest global rates recorded during the study period were found among subjects from Subsaharan Africa (238.51/100,000 inh.) and from Latin America (77.77/100,000 inh.), while the rate for those born in Spain was only 11.9/100,000 inhabitants.

**Table 1:** Epidemiological characteristics of cases diagnosed of HIV, syphilis, gonorrhea and lymphogranuloma venereum (LGV), by sexual conduct and sex. Barcelona, 2007–2011

Epidemiological characteristics	HIV			Syphilis			Gonorrhea			LGV
	Men		Women	Men		Women	Men		Women	Men
	1388		172	1033		91	1031		130	150
	MSM*	MSW**	WSM***	MSM*	MSW**	WSM***	MSM*	MSW**	WSM***	MSM*
	1096	228	172	834	112	91	528	244	130	143
Age										
Mean (SD)	34.7 (8.7)	38.6 (12.3)	36.6 (9.9)	36.4 (9.3)	37.3 (12.3)	36.4 (11)	32.7 (7.5)	31.8 (9.4)	31.2 (10)	37.1 (7.5)
Age group (%)										
15 – 24 years	120 (10.9)	21 (9.2)	18 (10.5)	49 (5.9)	15 (13.4)	11 (12.1)	57 (10.8)	51 (20.9)	28 (21.5)	3 (3.1)
25 – 34 years	503 (45.9)	80 (35.3)	67 (39)	343 (41.1)	35 (31.3)	33 (36.3)	282 (53.4)	120 (49.2)	61 (46.9)	54 (37.8)
35 – 44 years	346 (31.6)	70 (30.5)	52 (30.2)	311 (37.3)	35 (31.3)	31 (34.1)	150 (28.4)	46 (18.9)	24 (18.5)	65 (45.5)
45 years or older	127 (11.6)	57 (25)	35 (20.3)	131 (15.7)	27 (24.1)	16 (17.6)	39 (7.4)	27 (11.1)	16 (12.3)	21 (14.7)
Country of Birth (%)										
Spain	548 (50)	133 (58.3)	86 (50)	421 (50.5)	37 (33)	19 (20.9)	286 (54.2)	134 (54.9)	52 (40)	79 (55.2)
Western Europe, United States, Canada and Australia	131 (12)	8 (3.5)	7 (4.1)	124 (14.9)	7 (6.3)	3 (3.3)	89 (16.9)	13 (5.3)	12 (9.2)	25 (17.5)
Eastern Europe	22 (2)	18 (8.8)	6 (3.5)	18 (2.2)	7 (6.3)	9 (9.9)	13 (2.5)	8 (3.3)	3 (2.3)	2 (1.4)
Latin America	369 (33.7)	43 (18.9)	41 (23.8)	243 (29.1)	31 (27.7)	51 (56)	116 (22)	50 (20.5)	54 (41.5)	35 (24.5)
Sub–saharan Afrika	6 (0.5)	11 (4.8)	25 (14.5)	1 (0.1)	1 (1)	2 (2.2)	1 (0.2)	10 (4.1)	1 (0.8)	0 (0)
Other	19 (1.7)	13 (5.7)	7 (4.1)	17 (2.1)	27 (23.2)	7 (7.7)	10 (1.9)	22 (9)	3 (2.3)	1 (0.7)
Missing	1 (0.1)	0 (0)	0 (0)	10 (1.2)	3 (2.6)	0 (0)	13 (2.5)	7 (2.6)	5 (3.8)	1 (0.7)
Condom use										
Yes				201 (24.1)	12 (10.7)	11 (15.5)	96 (18.2)	31 (12.7)	37 (31.6)	12 (8.4)
No				425 (51)	67 (59.8)	37 (40.7)	348 (65.9)	167 (68.4)	65 (58.1)	98 (68.5)
Missing				208 (24.9)	33 (29.5)	43 (47.3)	84 (15.9)	46 (18.9)	25 (19.3)	33 (23.1)
Number of sexual partners										
Median (min – max)				10 (1 – 400)	2 (1 – 60)	1 (1 – 100)	9 (1 – 210)	3 (1 – 50)	2 (1–100)	15 (1 – 100)

*MSM: Men who have sex with men

**MSW: Men who have sex with women

***WSM: Women who have sex with men

*SD* Standard deviation

**Fig. 1:**
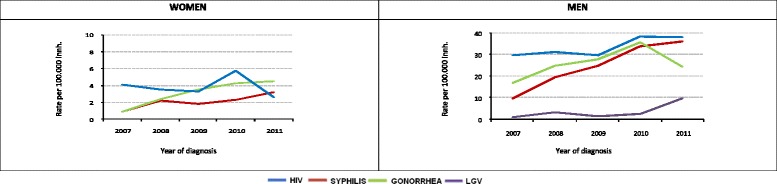
Incidence of syphilis, gonorrhea, lymphogranuloma venereum and HIV by sex. Barcelona, 2007–2011

Overall, a significant 10 % rise in the rate was observed, from 20/100,000 inh. in 2007 to 22/100,000 inh. in 2011 (*p* = 0.015). Among men, there was an increase of 25 %, while among women a decline of 38 % was observed (Fig. 1).

**Fig. 2:**
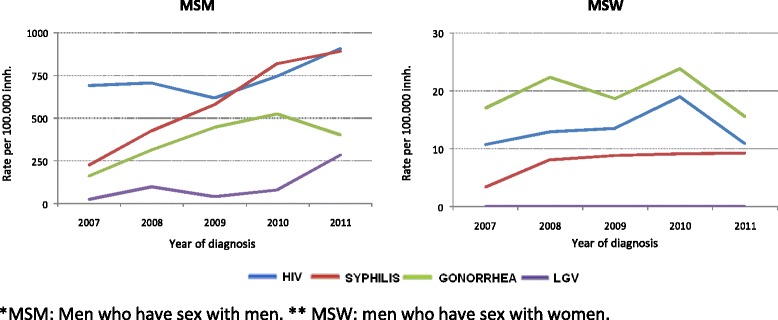
Incidence of syphilis, gonorrhea, lymphogranuloma venereum and HIV in men by sexual conduct. Barcelona, 2007–2011

MSM accounted for the highest proportion of cases (66.8 % of the total), as well as the highest rates, with an increase of 31 % over the study period (from 692.67/100,000 to 909.88/100,000 inh.) (*p* < 0.001) (Fig. 2).

### Syphilis

A total of 1124 cases were detected, 91.9 % (*n* = 1033) being men, among whom 80.7 % (834) declared themselves to be MSM (6.7 % of all MSM had sex with both males and females and 93.3 % had sex with men only). The MSM had a mean age of 36.4 years (SD:9.3), MSW had a mean age of 37.3 years (SD:12.3), and the WSM of 36.4 years (SD:11); differences between the groups were not significant (*p* = 0.37). Regarding country of birth, 50.5 % (421) of MSM and 33 % (37) of MSW had been born in Spain, while 56 % (*n* = 51) of WSM had been born in Latin America (Table 1). However, the highest rates were found among cases born in Subsaharan Africa (111.65/100,000 inh.) and in Latin America (57.91/100,000 inh.) both well above the rate for those born in Spain (7.9/100,000 inh.).

Over the period studied, the rate rose by 267 %, from 6/100,000 to 22/100,000 inhabitants (*p* < 0,001). Among men there was a rise of 273 %, in women of 240 % (Fig. 1).

**Fig. 3:**
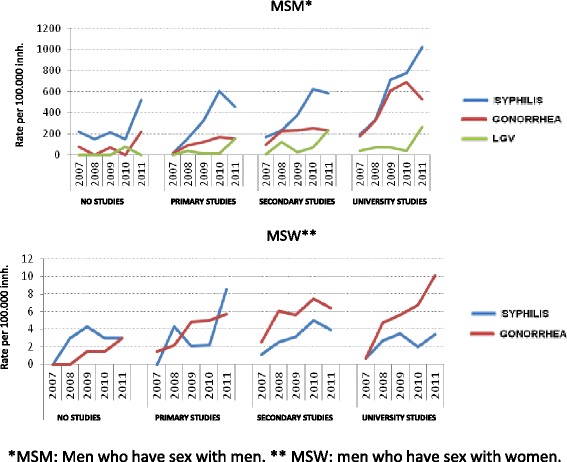
Incidence of syphilis, gonorrhea, lymphogranuloma venereum in men by sexual conduct and education level. Barcelona, 2007–2011

The MSM accounted for the highest proportion of cases (74.2 % of the total), as well as the highest rates, a rise of 296 % being observed over the period (from 224.9/100,000 to 891.97/100,000 inh.) (*p* < 0.001). Thus, in 2011 their rate was 175 times higher than that of WSM (5.11/100,000 inh.) and 96 times higher than MSW (9.29/100,000 inh.) (Fig. 2). In relation to educational level, MSM with a university education presented the highest rates, ranging from 196.3/100,000 inh. in 2007 to 1020.8/100,000 inh. in 2011, representing an increase of 420 % in this group (*p* < 0.001) (Fig. 3).

Regarding use of a condom the last time they had sex, 24.1 % of MSM, 10.7 % of MSW and 15.5 % of WSM reported doing so. The median number of sexual partners in the last 12 months was 10 among MSM (min:1; max:400), 2 among MSW (min: 1; max: 60), and 1 among WSM (min: 1; max: 100), this information being available in 75.8 % of cases (Table 1).

### Gonorrhea

A total of 1161 cases were detected, 88.8 % (*n* = 1031) of whom were men and of these 51.2 % (528) declared themselves to be MSM (8.1 % of all MSM had sex with both males and females and 91.9 % had sex with men only). The mean age of the MSM was 32.7 years (SD:7.5), of MSW was 31.8 (SD:9.4) and of WSM was 31.2 (SD:10), with no significant differences between groups (*p* = 0.21). Regarding country of birth, 54.2 % (286) of the MSM and 54.9 % (134) of the MSW were born in Spain, while 41.5 % (54) of the WSM were born in Latin America (Table 1). However, Latin America was the region of birth which presented the highest incidence rates (44.61/100,000 inh.) followed by Eastern Europe (40.66/100,000 inh.), both rates being higher than that among subjects born in Spain (9.5/100,000 inh.).

During the study period, a significant increase of 50 % was observed in the rates, which rose from 10/100,000 inh. to 15/100,000 inh.(*p* < 0.0001). The increase among men was therefore 45 %, while among women it was 353 % (Fig. 1).

The MSM group accounted for 45.5 % of all recorded cases and present the highest incidence rates, which rose by 146 % over the study period (from 164.24/100,000 to 404.79/100,000 inh.) (*p* < 0.01) (Fig. 2). Hence, in 2011 their rate was 60 times higher than that of WSM (6.75/100,000 inh.), and 26 times higher than that of MSW (15.62/100,000 inh.) (Fig. 2). Regarding educational level, the MSM with a university education presented the highest rates, which ranged from 176.7/100,000 inh. in 2007 to 530.1/100,000 inh. in 2011, representing an increase of 200 % (*p* < 0.001) (Fig. 3).

In relation to whether they used a condom the last time they had sex, 18.2 % of MSM, 12.7 % of MSW and 31.6 % of WSM report doing so. The median number of sexual partners in the last 12 months was 9 among MSM (min: 1; max: 1000), 3 among MSW (min: 1;max: 50) and 2 among WSM (min: 1;max: 100), this information being available in 78.6 % of cases (Table 1).

### Lymphogranuloma venereum

A total of 150 cases were detected, all corresponding to men, 95.3 % of whom declared themselves to be MSM (0.7 % of all MSM had sex with both males and females and 99.3 % had sex with men only). In seven cases this information was not available. The mean age was 37 years; 56.7 % of cases were born in Spain (Table 1); overall incidence rate was 1.3/100,000 inhabitants. The highest incidence rate corresponded to cases born in Western Europe (8.13/100,000 inh.) follow by those born in Latin America (5.89/100,000 inh.). During the study period there was an increase in the incidence among MSM of 1032 % (from 24.99/100,000 inh. in 2007 to 282.99/100,000 inh. in 2011) (Fig. 2).

In relation to educational level, cases with a university education presented the highest rates, which ranged from 39.3/100,000 inh. in 2007 to 265/100,000 inh. in 2011, representing an increase of 574 % (*p* < 0.001) (Fig. 3).

Regarding whether they used a condom the last time they had sex, 8.4 % of cases reported doing so. Information on the number of sexual partners in the last 12 months was available for 45.9 % (*n* = 69) cases, the median number among MSM being 15 (min: 1; max: 100) (Table 1).

## Discussion

With respect to other cities in developed countries, in 2010 our HIV incidence rates were higher than in London (15/100,000 inh.) [15] and lower than those in New York (42.6/100,000 inh.) [16]. In the case of syphilis, our incidence rates were lower (London: 14/100,000 inh.; New York: 130/100,000 inh) [4, 6, 7, 17]. However, with respect to Spain as a whole, our incidence rates were 3.5 times higher for both syphilis and gonorrhea [18].

The ratio of gonorrhea to syphilis cases was changing over time, as seen in the rest of Europe where the ratio had been decreasing [5]. In Barcelona, in 2007 the ratio was 1.6, in 2008 was 1.2, in 2009 was 1.1, in 2010 was 1 and in 2011 was 0.7. This decrease was clearly related to sexual conduct variable due to an increase in the proportion of MSM was observed over the five years and the syphilis had a higher proportion of MSM than gonorrhea.

The group of MSM were the most affected, as found in other large cities of Western countries [6, 7]. The rise in STI incidence could be influenced by a rise in risky sexual conduct, including: an increase in number of sexual partners, subgroups engaging in more risky sexual practices and a higher frequency of unprotected sexual practices [8, 19–21]. This had been observed in recent years with epidemics such as that of LGV [22] and outbreaks of hepatitis [23], which were indicative of a rise in risky sexual conduct in this group. This fact may be explained according to various sociological theories, which suggest that risky sexual conduct among MSM could constitute a mechanism to cope with or escape from stress resulting from the perception of social rejection [24, 25].

Another possible reason that justifies the observed increase is the failure of HIV prevention strategies (e.g. sero-sorting/positioning, treatment as prevention, pre/post-exposure prophylaxis, etc.). There is still some way to go in identifying which of these upstream interventions are effective, how they may be implemented within or alongside existing health care systems, and what impact, if any, they are likely to have on STD transmission. Today, effective HIV prevention requires a combination of behavioral, biomedical, and structural intervention strategies.

In regard to educational level, MSM with a university education were the most affected group. At national level it had also been observed that people with more education (secondary and university studies) presented the highest incidence rates [26]. Possible reasons which could justify this result included: greater access to health services by the better educated population, over-estimation of the university population, being a self-reported variable and/or that the population with more resources was more careless about the problem of STI due to the efficiency of antiretroviral therapies and thus had less fear of HIV.

With respect to place of birth, it was notable that half the STI cases occurred among foreigners. For this reason it was necessary to ensure access to health services among this particularly vulnerable group.

Among the limitations of the present study, one was the possibility of an over-estimation of university level education, since the variable was self-reported as already mentioned. However, we believed that the rates were close to the true values, as they were similar to those in the rest of the country [26]. Another limitation of the study was the large number of missing values in two self-reported variables: number of sexual partners and condom use. Finally, we must mention the possible existence of recall bias in the self-reported variables (educational level, number of sexual partners, condom use and sexual conduct).

## Conclusions

An increase in cases of STI was observed in the city of Barcelona over the period studied, particularly cases of syphilis and LGV. And the increase in STI was observed in all categories of sexual conduct, except HIV-infected WSM for which a reduction during the study period was observed.

MSM constituted the group most affected by infections of this type, particularly those with a university education. This suggests that although people were informed about sexually transmitted infections, they did not take sufficient preventive measures to avoid them, meaning that a change is probably necessary in the different preventive messages that they currently receive.

This study shows that STI must be a priority in public health. In Barcelona, different preventive programs have been developed to try to reduce the incidence of STIs, such as a sauna program and a partner notification program [27]. We have also created 3 specialized units of STI to increase early diagnosis and reduce damage and in recent years the collaboration between public health services and community associations has enhanced for early diagnosis of STIs. However, it is still necessary to increase the public health measures to achieve a better result, such as the integrated management of STI cases (diagnostic circuit, identification and referral), improve the mechanism of notificable diseases and integrate a partner notification program for new STI cases.

MSM are still at risk for HIV infection despite 3 decades of prevention efforts, the rise in HIV and other STI needs to be reversed soon [28]. Continuing to monitor changes in the epidemiology of STI, and identifying the most affected groups should permit redesigning preventive programs, with the goal of finding the most efficient ways to reach these populations. In our setting, we consider that priority actions should be the promotion of measures to improve condom use, health education, treatment and particularly early diagnosis by investigating sexual partners due to the effectiveness in reducing morbidity and mortality [29, 30].

## Abbreviations

STI: Sexually transmitted infections
MSM: Men who have sex with men
HAART: Highly active antiretroviral therapy
LGV: Lymphogranuloma Venereum
MSW: Men who only have sex with women
WSM: Women who have sex with men
CI: Confidence Intervals
